# Recombinant Human Thrombopoietin Accelerates the Recovery of Platelet in Patients With Lower-Risk Myelodysplastic Syndrome: A Proof-of-Concept Study

**DOI:** 10.3389/fonc.2021.721764

**Published:** 2021-10-28

**Authors:** Yuan Yang, Zengwei Tang, Jiang Ji, Chen Yang, Miao Chen, Bing Han

**Affiliations:** ^1^ Department of Hematology, Peking Union Medical College Hospital, Chinese Academy of Medical Sciences and Peking Union Medical College, Beijing, China; ^2^ Department of Hepatobiliary and Pancreatic Surgery, The First Affiliated Hospital, School of Medicine, Zhejiang University, Hangzhou, China

**Keywords:** lower-risk myelodysplastic syndrome, recombinant human thrombopoietin, hematologic response, adverse events, platelet response

## Abstract

**Aim:**

The effect of recombinant human thrombopoietin (rhTPO) is largely unknown in lower-risk myelodysplastic syndrome (LR-MDS). This study aimed at investigating the safety and efficacy of rhTPO in patients with LR-MDS.

**Methods:**

LR-MDS patients receiving stanozolol (2 mg, t.i.d.) and supportive care alone (non-rhTPO) or additional rhTPO were enrolled in this study prospectively. rhTPO was given at 15,000 U (q.d.) for 7 days/month for at least 3 months. Patients stopped rhTPO if the platelet count was higher than 50 × 10^9^/L or had no effects after 3 months of treatment. The overall response (OR), complete response (CR), platelet response, side effects, clone evolution, and clinical outcome were evaluated.

**Result:**

Thirty-five patients were enrolled: 20 (57.1%) patients in the rhTPO group and 15 (42.9%) patients in the non-rhTPO group. The demographic and baseline characteristics were balanced between the two groups. Platelet response was higher at 1 and 2 months as compared with that in the non-rhTPO group (*p* = 0.006 and *p* = 0.001, respectively). Meanwhile, the rhTPO group had a shorter time to achieve a platelet transfusion-free state compared with the non-rhTPO group (*p* = 0.034). Hematologic response was higher at 1 and 2 months compared with that in the non-rhTPO group (*p* = 0.006 and *p* = 0.001, respectively). There was no significant difference in the overall response or complete response at 1, 2, 3, 6, and 12 months between the two groups. One patient in the rhTPO group evolved into higher-risk MDS at 9 months. No significant difference in disease progression, infection, gastrointestinal disorders, or drug-related liver/renal injuries was found between the two groups (*p* > 0.05).

**Conclusion:**

Adding short-term rhTPO can accelerate the early platelet response and decrease platelet transfusion, with no obvious side effects.

**Clinical Trial Registration:**

https://clinicaltrials.gov/ct2/show/NCT04324060?cond=NCT04324060&draw=2, identifier NCT04324060

## Introduction

Myelodysplastic syndrome (MDS), a heterogeneous disease of clonal myeloid disorders, is characterized by ineffective hematopoiesis, bone marrow dysplasia, and peripheral cytopenia(s), which has a high tendency of transforming into acute myeloid leukemia (AML) (1, 2). More than 60% of MDS patients are lower risk (LR-MDS), which are characterized by the presence of dysplasia, low bone marrow blast percentage, low number and depth of cytopenia(s), and relatively good risk karyotypic and molecular abnormalities. A score of ≤3.5 on the Revised International Prognostic Scoring System classifies patients as lower-risk MDS (3, 4).

The prognosis of MDS is correlated with the degree of cytopenia(s), bone marrow blast percentage, and the presence of specific cytogenetic abnormalities (5). MDS patients mainly die either from complications (including infections and bleeding) or from transformation to AML (6).

Some patients with LR-MDS can present with an isolated thrombocytopenia, and the incidence of life-threatening thrombocytopenia (platelet count <20 × 10 (7)/L) has been reported in approximately 12% of patients with LR-MDS (8, 9). Platelet transfusions are not durable and are highly associated with allergic transfusion reactions, transmission of bacterial and viral infections, and transfusion-related acute lung injury (7). Alloimmunization, most common in patients with MDS, ultimately renders platelet transfusions ineffective (9, 10).

Thrombopoietin (TPO) is the primary regulator of megakaryocyte development and platelet production through binding of the TPO receptor, c-MPL (11). Accordingly, thrombopoietin receptor agonists (TPO-RAs), including eltrombopag (EPAG), romiplostim, and avatrombopag, have been approved for the treatment of chronic immune thrombocytopenia (ITP) and/or aplastic anemia (AA) (12–14). In MDS, monotherapy with thrombopoietin agonists has been tested in two studies, in which increased platelet counts were seen in nearly 50% of the patients (15, 16). LR-MDS patients receiving romiplostim therapy had an apparently increased risk of AML progression (17).

Furthermore, transient elevations of circulating blasts were observed in ~10% of patients, for which close monitoring is recommended, as well as avoidance of TPO-RA use in MDS patients with excess blasts (>5%) (17). In order to maintain the effects, TPO-RAs have to be used for long term, which further causes huge economic burden and a higher risk of bone marrow fibrosis and leukemia transformation. All of these concerns highlight the importance of finding new TPO-RAs for MDS patients.

Recombinant human thrombopoietin (rhTPO), a glycosylated full-length peptide TPO produced by 3SBIO (Shenyang, China), has shown a high response rate (60.3%–66.7%) in corticosteroid-resistant or relapsed ITP patients (18–21). Although patients developed transient anti-TPO antibodies after rhTPO treatment, the antibodies did not have the activity of neutralizing endogenous TPO (22).

However, the therapy clinical outcomes of rhTPO in LR-MDS patients are rarely reported. Therefore, we performed this proof-of-concept study to investigate the safety and efficacy of rhTPO in patients with LR-MDS.

## Methods

### Patients and Eligibility

This is a proof-of-concept study. Patients newly diagnosed with LR-MDS at Peking Union Medical College Hospital between December 2018 and March 2020 were included, in accordance with the following criteria: 1) ≥18 years old; 2) diagnosed with LR-MDS based on standard criteria (3); 3) blast cells <5% in the bone marrow (BM) and <1% without Auer’s body and blast cells <1% in peripheral blood; 4) platelet counts <30 × 10^9^/L and/or platelet transfusion dependence; and 5) patients with adequate renal and hepatic functions [alanine transaminase (ALT)/aspartate transaminase (AST) within three times the normal upper limit, total bilirubin (TBIL) within two times the normal upper limit, and creatinine within two times the normal upper limit]. The exclusion criteria were as follows: 1) patients with a history of leukemia or stem cell transplantation, treatment-related MDS, or malignancies; 2) patients with active or uncontrolled infections, uncontrolled cardiovascular disease, or a history of arterial or venous thrombosis within the past year; 3) patients who had received any other thrombopoietic growth factors prior to rhTPO therapy; and 4) patients with MDS secondary myelofibrosis. Patients were randomized into two groups: the rhTPO group and the non-rhTPO group. This study design was approved by the Committees for the Ethical Review of Research at Peking Union Medical college Hospital. The clinical trial was registered at clinicaltrial.gov (NCT04324060). The corresponding web link is as follows: https://clinicaltrials.gov/ct2/show/NCT04324060?cond=NCT04324060&draw=2&rank=1.

### Treatment and Follow-Up Regimen

For the rhTPO group, patients were treated with 2 mg (t.i.d.) stanozolol for at least 1 year, supportive treatment, and rhTPO (recombinant human thrombopoietin injection; SanSheng Pharmaceutical, Shenyang, China) of 15,000 U subcutaneously (q.d.) for 7 days and continued for at least 3 months if the platelet count did not reach 50 × 10^9^/L for three consecutive days. For the non-rhTPO group, patients were treated with 2 mg (t.i.d.) stanozolol for at least 1 year only and supportive treatment. Supportive treatment included transfusion, recombinant human erythropoietin (rhEPO; SanSheng Pharmaceutical, Shenyang, China) of 10,000 U three times/week for at least 3 months if hemoglobin was <110 g/L and/or granulocyte colony stimulating factor (G-CSF; 5 μ g kg^−1^ day^−1^) if neutrophil was <0.5 × 10^9^/L.

The demographic characteristics of patients were recorded, which included present and past history, physical examination data, tests for complete blood cell count (CBC), and biochemistry parameters such as liver and kidney function, serum ferritin (SF), bone marrow smear, bone marrow biopsy, chromosome, MDS/myeloproliferative neoplasm (MPN) gene mutations, if possible, and paroxysmal nocturnal hemoglobinuria (PNH) clone size. Patients in each group were followed up monthly after treatment for at least 6 months and then every 3 months for at least 1 year and were evaluated for response and side effects. Routine lab tests were repeated regularly, and the bone marrow smear, bone marrow biopsy, and chromosome tests were repeated when necessary or every 6 months regularly for at least 1 year. The final response and disease outcomes were recorded at the end of the follow-up.

### Response Criteria

Efficacy was evaluated using the International Working Group (IWG) criteria during the treatment period (23). Platelet counts were not taken into consideration for efficacy evaluation if platelet transfusion was given within 3 days of evaluation. Platelet response was defined as an absolute increase of ≥30 × 10^9^/L for patients starting with >20 × 10^9^/L platelets or an increase from <20 × 10^9^/L to >20 × 10^9^/L and by at least 100%. Neutrophil response was defined as at least a 100% increase and an absolute increase >0.5 × 10^9^/L. Erythroid response was defined as a hemoglobin increase ≥15 g/L or a relevant reduction of red blood cell (RBC) transfusion by at least 50% compared with the pretreatment RBC transfusion in the previous 8 weeks. Hematologic response referred to specific responses meeting the criteria of platelet, neutrophil, or erythroid response.

Complete response (CR) was defined by the presence of ≤5% blasts in the bone marrow without indication of dysplasia, a platelet count increase ≥100 × 10^9^/L, a hemoglobin increase ≥110 g/L, an absolute neutrophil count (ANC) increase >1.0 × 10^9^/L, and no blast cell in the peripheral blood. For partial response (PR), responding patients must fit all CR criteria, but with bone marrow blasts reduced by ≥50% over the pretreatment value (23).

Safety was assessed from the incidence and severity of adverse events and classified according to the National Cancer Institute Common Toxicity Criteria for Adverse Events, version 5.0 (24). Bleeding episodes were recorded as adverse events, and bleeding adverse events grade 2 or higher severity were considered clinically significant. The definition of disease progression was ≥50% increase in blasts and at least <50% decrement from maximum response in platelets or a reduction in hemoglobin by ≥20 g/L or transfusion dependence (20, 22).

### Sample Size and Statistics Analysis

We assumed that the platelet response in patients treated with rhTPO might be 1.3 times that of the controls (based on the experience of rhTPO in newly diagnosed adult primary ITP) (21). Power calculation indicated that if we want to have an 85% chance of rejecting the hypothesis with no difference at the level of 0.05, we needed 62 patients in each group. At the intermediate analysis, when we had recruited about 40 patients, we found that those treated with rhTPO already had significantly higher platelet response than those without rhTPO treatment. To avoid the hemorrhage-associated events for patients without rhTPO treatment, we stopped recruiting participants afterwards.

The summary statistics for patient demographics and laboratory measurements were presented using the median and range or percentage. Fisher’s exact test or the chi-square test and Student’s *t*-test were used to calculate the significance of the difference between the rhTPO and non-rhTPO groups. Covariate effects on the platelet response rate were evaluated using univariable logistic regression, with statistical inference presented using the corresponding standard errors. A *p*-value <0.05 was considered statistically significant. All statistical analyses were performed using SPSS (version 21.0).

## Results

### Basic Patient Characteristics

**Figure 1:**
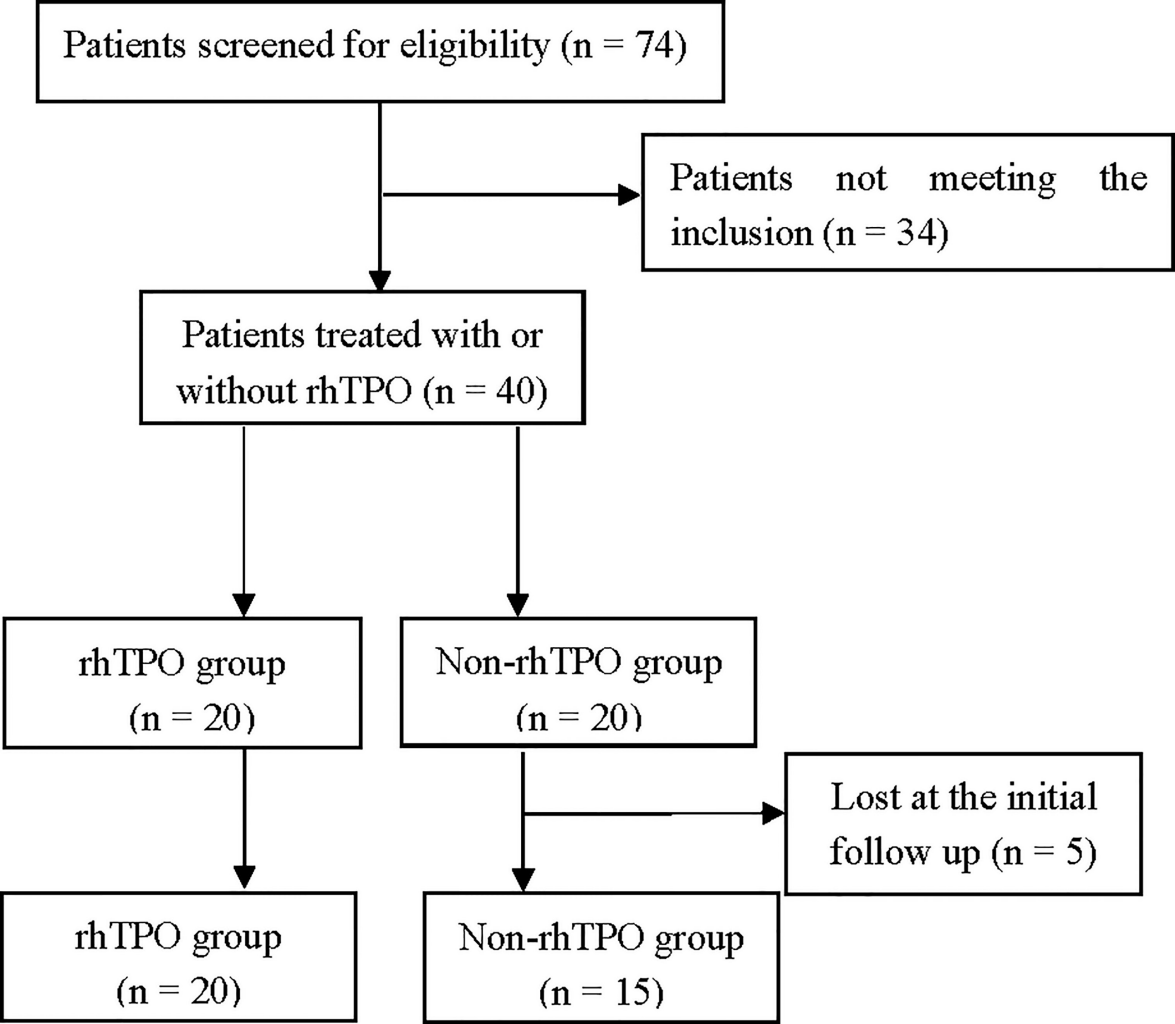
Trial profile. Seventy-four patients were screened for eligibility; 34 patients were excluded for not meeting the inclusion criteria. Forty patients were ultimately enrolled, of whom 20 were assigned to the recombinant human thrombopoietin (rhTPO) group and the other 20 to the non-rhTPO group. Five patients in the non-rhTPO group were lost within 3 months at the initial follow-up and were excluded from the final analysis. The remaining 35 patients completed the designed treatment.

Seventy-four patients were screened for eligibility, and 34 patients were excluded for not meeting the inclusion criteria. Forty patients were ultimately enrolled, of whom 20 were assigned to the rhTPO group and the other 20 to the non-rhTPO group (**Figure 1**). Five patients in the non-rhTPO group were lost within 3 months at the initial follow-up and were excluded from the final analysis. The remaining 35 patients completed the designed treatment and were followed for at least 1 year. The median follow-up times were 14 months (12–24 months) in the rhTPO group and 15 months (12–20 months) in the non-rhTPO group.

For the rhTPO group (*n* = 20), the median age was 57 years (range = 41–91 years) and 11 patients (55%) were males. Eleven patients (55%) were assessed as low risk and the remaining 9 (45%) patients were determined as intermediate-1 risk based on the Revised International Prognostic Scoring System (IPSS-R) risk classification. Among them, 4 (20%) patients had refractory cytopenia with unilineage dysplasia, 14 (70%) had refractory cytopenia with multilineage dysplasia, and 2 (10%) had myelodysplastic syndrome—unclassified. For the non-rhTPO group (*n* = 15), the median age was 54 years (range = 46–82 years) and 6 (40%) patients were males. Nine (60%) patients were low risk and 6 (40%) patients were determined as having intermediate-1 risk MDS. Four (26.7%) patients had refractory cytopenia with unilineage dysplasia, 8 (53.3%) patients had refractory cytopenia with multilineage dysplasia, and 3 (20%) patients had myelodysplastic syndrome—unclassified. The median hemoglobin, neutrophil and platelet counts were 82g/L (range = 49–132), 1.77 × 10 (7)/L (range = 0.12–6.63), and 17 × 10^9^/L (range = 6-29) for patients in the rhTPO group and were 89 g/L (range = 45–115), 1.52 × 10^9^/L (range = 0.31–4.50), and 18 × 10^9^/L (range = 7–29) for patients in the non-rhTPO group, respectively.

An abnormal karyotype was shown by 3 (15%) patients [trisomy 8 and del(12p) and del (20q)] in the rhTPO group and by 2 (13.3%) patients [trisomy 19 and del(20q)] in the non-rhTPO group, which was based on the results from the chromosome banding analysis at the diagnostic workup.

Furthermore, according to the IPSS cytogenetic risk classification, 4 (20%) patients from the rhTPO group and 3 (20%) patients from the non-rhTPO group were classified as intermediate risk (*p* = 1.000); the remaining patients (80%) in the two groups were good risk. There was no difference in concomitant treatment during the study period. All baseline characteristics were similar between the two groups. Detailed information is outlined in **Table 1**.

**Table 1:** Patient characteristics at baseline.

Baseline characteristics	Total (*n* = 35)	rhTPO group (*n* = 20)	Non-rhTPO group (*n* = 15)	*p*-value
Age (years), median (range)	55 (41–91)	57 (41–91)	54 (46–82)	0.640
Male/female, *n* (%)	17 (48.7)/18 (51.3)	11 (55)/9 (45)	6 (40)/9 (60)	0.287
WHO classification, *n* (%)				
RCUD	8 (22.8)	4 (20)	4 (26.7)	0.994
RCMD	22 (62.9)	14 (70)	8 (53.3)	
MDS-U	5 (14.3)	2 (10)	3 (20)	
IPSS-R risk, *n* (%)				
Low	20 (57.1)	11 (55)	9 (60)	0.999
Intermediate-1	15 (42.9)	9 (45)	6 (40)	
Neutrophil count (10^9^/L), median (range)	1.66 (0.12–6.63)	1.77 (0.12–6.63)	1.52 (0.31–4.50)	0.807
Platelet count (10^9^/L), median (range)	19 (6–29)	17 (6–29)	18 (7–29)	0.478
Hemoglobin (g/dl), median (range)	87 (45–132)	82 (49–132)	89 (45–115)	0.816
ALT (U/L), median (range)	14 (10–22)	7 (15–21)	12 (10–22)	0.230
AST (U/L), median (range)	16 (8–24)	16 (11–24)	15 (8–23)	0.832
TBIL (µmol/L), median (range)	14.3 (8.2–17.4)	13.3 (8.2–16.6)	15.3 (11.7–17.4)	0.226
Cr (µmol/L), median (range)	71.0 (50–91)	70.5 (50.0–91.0)	78.0 (54.0–89.0)	0.577
Serum ferritin (g/dl), median (range)	448 (142–2192)	436 (151–2192)	448 (142–1350)	0.201
Albumin (g/L), median (range)	40.0 (32–46)	40.5 (33–46)	40.0 (32–44)	0.886
Lactate dehydrogenase (IU/L), median (range)	231 (146–929)	243 (146–929)	219 (167–461)	0.534
rhEPO, median	8 (22.9)	4 (20)	4 (26.7)	0.859
G-CSF, median	12 (34.3)	7 (35)	5 (33.3)	0.975
Follow-up (months), median (range)	15 (6–24)	14 (6–24)	15 (12–20)	0.103

rhTPO, recombinant human thrombopoietin; WHO, World Health Organization; RCUD, refractory cytopenia with unilineage dysplasia; RCMD, refractory cytopenia with multilineage dysplasia; MDS-U, myelodysplastic syndrome—unclassifiable; IPSS-R, Revised International Prognostic Scoring System; ALT, alanine transaminase; AST, aspartate transaminase; TBIL, total bilirubin; Cr, creatinine; MDS, myelodysplastic syndrome; PNH, paroxysmal nocturnal hemoglobinuria; rhEPO, recombinant human erythropoietin; G-CSF, granulocyte colony stimulating factor.

### Response

**Figure 2:**
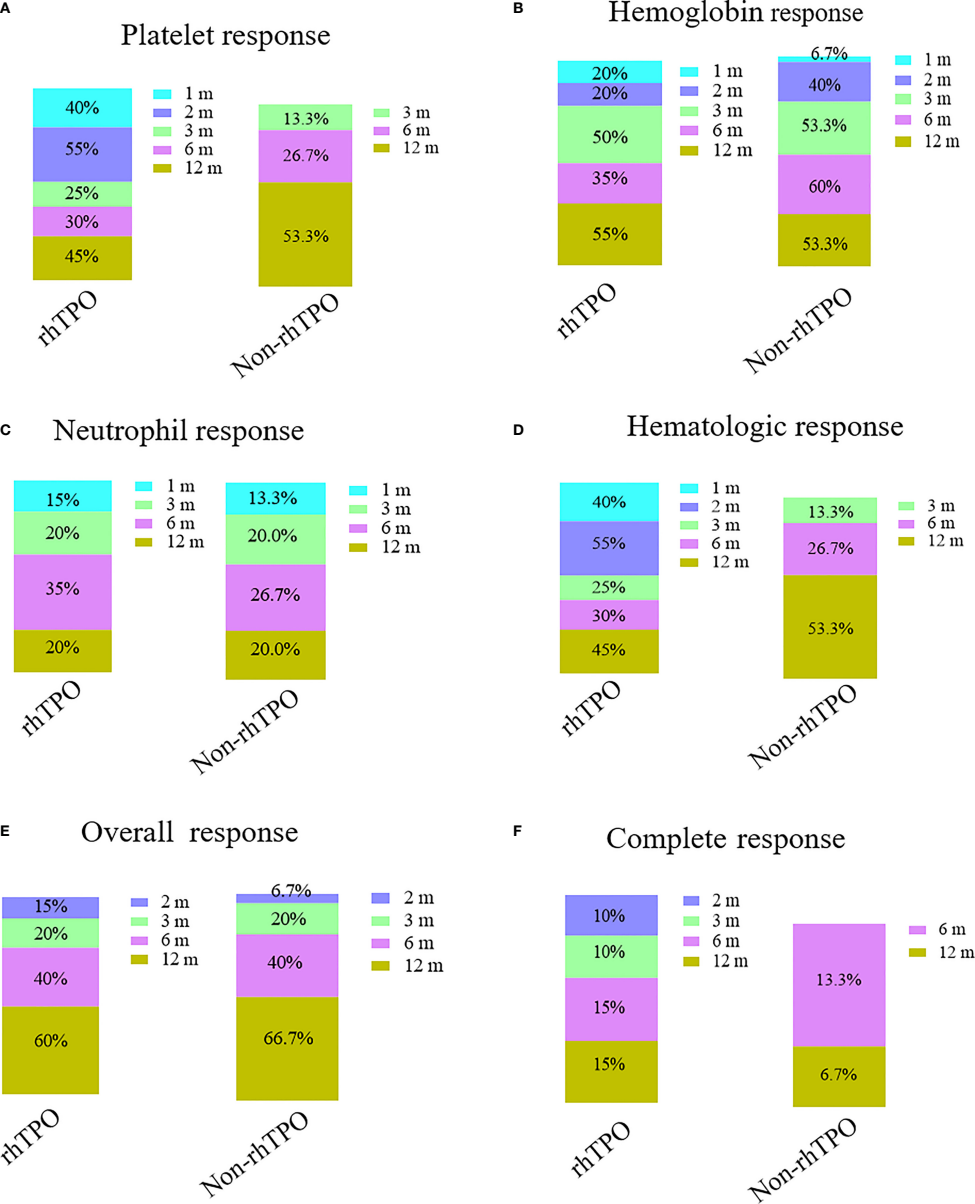
Bar plots showing the therapy response rates at different time points in the recombinant human thrombopoietin (rhTPO) group and the non-rhTPO group. Platelet response **(A)**, hemoglobin response **(B)**, neutrophil response **(C)**, hematologic response **(D)**, complete response **(E)**, and overall response **(F)** rates at 1, 2, 3, 6, and 12 months in the rhTPO and non-rhTPO groups.

In the rhTPO group, three patients discontinued the treatment after 1 month of therapy due to platelet counts higher than 50 × 10^9^/L. Four patients stopped treatment because of platelet counts higher than 50 × 10^9^/L after 2 months of therapy; 13 patients were treated with rhTPO for 3 months. The median therapy duration for rhTPO treatment was 3 months (range = 1–3 months). The therapy response at 1, 2, 3, 6, and 12 months is summarized in **Figure 2**. The overall response rates (ORRs) at 1, 2, 3, 6, and 12 months were 0%, 15%, 20%, 40%, and 60% for the rhTPO group and 0%, 6.7%, 20%, 40%, and 66.7% for the non-rhTPO group, respectively (*p* > 0.05). Meanwhile, the complete response rates (CRRs) were 0%, 10%, 10%, 15%, and 15% for the rhTPO group and 0%, 0%, 0%, 6.7%, and 13.3% for the non-rhTPO group at 1, 2, 3, 6, and 12 months, respectively (*p* > 0.05). A significant hematologic response was observed at 1 and 2 months after treatment (40.0% *vs*. 0%, *p* = 0.006; 55.0% *vs*. 0%, *p* = 0.001, respectively). However, the hematologic response at 3, 6, and 12 months was not statistically different between the two groups (*p* > 0.05).

Although there was no significant difference at 1, 2, 3, 6, and 12 months for the hemoglobin and neutrophil response between the two groups, a significant platelet response was observed at 1 and 2 months after treatment (40% *vs*. 0%, *p* = 0.006; 55.0% *vs*. 0%, *p* = 0.001, respectively). However, the platelet response at 3, 6, and 12 months was not statistically different between the two groups (*p* > 0.05).

Similarly, the rhTPO group had a shorter time to achieve a platelet transfusion-free state compared with the non-rhTPO [2 (range = 1–6) *vs*. 4.5 (range = 2–12) months, respectively, *p* = 0.034], although the rate of platelet transfusion independence at 1 year was not significantly different between the two groups (14/20 *vs*. 10/15, *p* = 0.976).

No severe bleeding events were observed in either group for the first 3 months after treatment. However, for the rhTPO group, one patient developed obvious skin petechiae at 5 months, which disappeared after platelet transfusion; another patient had intracranial hemorrhage (ICH) at 6 months after rhTPO discontinuation and died later. For the non-rhTPO group, one patient developed hematuria at 7 months, but improved after platelet transfusion.

With compatible baseline values, the megakaryocyte counts were 11 (range = 0–38) and 9 (range = 0–32) at 6 months for patients in the rhTPO and non-rhTPO group, respectively (*p* = 0.761). Meanwhile, there was also no increase in reticulin or fibrosis in any examined bone marrow after 6 and 12 months of treatment in either group.

Due to the small number of patients, we did not find positive predictors for hematologic response or platelet response when we included factors such as age, baseline hemoglobin, ANC, platelet level, lactate dehydrogenase (LDH), and SF (*p* > 0.05; **Table 2**).

**Table 2:** Univariable logistic model for platelet response.

Variable	Response at 1 month			Response at 2 months		
	Coefficient (*β*)	SE	*p*-value	Coefficient (*β*)	SE	*p*-value
Age	0.060	0.035	0.085	0.006	0.007	0.415
Platelet	−0.117	0.132	0.376	−0.004	0.024	0.856
Hemoglobin	−0.021	0.034	0.524	0.001	0.005	0.775
ANC	0.294	0.609	0.629	0.050	0.183	0.786
Serum ferritin	−0.002	0.002	0.253	−0.001	0.001	0.150
LDH	0.002	0.009	0.057	−0.001	0.002	0.414

ANC, absolute neutrophil count; LD, lactate dehydrogenase; SE, standard error.

### Final Outcome

For the rhTPO group, 3 (15%) patients had disease progression with a median time of 9 months (range = 6–9 months) (**Table 3**). One patient who presented with IPSS-R intermediate-1 and normal karyotype at baseline, responded at 3 months, but the platelet counts declined after 9 months. She was found to have deletion 7q chromosome abnormality; her myeloblasts increased from <2% to 5% and she progressed to high-risk MDS. Platelets and ANC declined in another patient at 9 months and she became platelet transfusion-dependent. The remaining one patient who had no response to rhTPO therapy had a decreased platelet count to 2 × 10^9^/L and died from cerebral bleeding at 6 months after the rhTPO treatment. No other deaths were found during the study period for the rhTPO group.

**Table 3:** Clinical characteristics of patients who progressed during follow-up.

rhTPO group				Non-rhTPO group			
	Patient 1	Patient 2	Patient 3	Patient 4	Patient 5	Patient 6	Patient 6
Age (years)	41	58	67	46	62	52	48
Sex	M	M	F	F	F	F	M
R-IPSS	Int-1	Int-1	Int-1	Int-1	Low	Int-1	Low
Baseline							
Karyotype	46,XY [20]	46,XY [20]	46,XX [20]	46,XX [20]	46,XX [20]	46,XX [20]	46,XY [20]
Bone marrow blasts (%)	<5	<5	<2	<5	<5	<5	<2
ANC (×10^9^/μl)	1.25	2.22	0.3	1.77	1.81	0.77	4.5
Hemoglobin (g/L)	69	54	72	45	98	66	101
Platelets (×10^9^/L)	13	16	6	8	21	6	16
Disease progression							
Karyotype	46,XY [20]	46,XY, [20]	46,XX,-7 [20]	46,XX [20]	46,XX [20]	46,XX [20]	46,XY [20]
Bone marrow blasts (%)	<5	<2	5	<5	<5	<5	<2
ANC (×10^9^/μl)	0.77	3.66	0.56	1.96	2.81	1.77	4.5
Hemoglobin (g/L)	67	54	65	68	104	73	190
Platelets (×10^9^/L)	5	2	5	3	10	2	5
Time to progression (months)	9	6	9	9	6	7	6
Present status	Alive	Dead	Alive	Alive	Alive	Alive	Alive

rhTPO, recombinant human thrombopoietin; ANC, absolute neutrophil count; Int-1, intermediate 1; Int-2, intermediate 2.

For patients in the non-rhTPO group, 5 (33.3%) had disease progression at a median time of 7 months (range = 6–9 months; **Table 3**). However, none of them had increased blast cell percentage or advance in cytogenetic findings. Of these patients, four remained platelet transfusion-dependent and the other one patient presented with PNH clone at 6 months, but did not meet the IWG criteria for disease progression. No patients died during the follow-up.

### Safety

One patient in the rhTPO group who had no response to therapy had a grade 4 adverse event (ICH) and died. No other serious adverse events occurred in both groups (**Table 4**). Most of the adverse events were either grade 1 or 2. Fatigue (10%), infection (10.0%), drug-related liver injury (15.0%), and gastrointestinal disorders (20%) were the most common adverse events in the rhTPO group. Drug-related liver injury (13.3%), infection (13.3%), and gastrointestinal disorders (20%) were the main adverse events in the non-rhTPO group. No significant difference in the drug-related adverse events was found between the two groups (*p* > 0.05).

**Table 4:** Adverse events.

Adverse events (*n*, %)	All patients (*n* = 35)	rhTPO group (*n* = 20)	Non-rhTPO group (*n* = 15)	*p*-value
Infection rate	4 (11.4%)	2 (10.0%)	2 (13.3%)	0.980
Drug-related liver injury	5 (14.3%)	3 (15.0%)	2 (13.3%)	0.991
Drug-related renal injury	2 (5.7%)	1 (5.0%)	1 (6.7%)	0.979
Fatigue	3 (8.6%)	2 (10.0%)	1 (6.7%)	0.809
Gastrointestinal disorders (diarrhea, nausea, and abdominal pain)	7 (20.0%)	4 (20.0%)	3 (20%)	1.000
Intracranial hemorrhage	1 (2.6%)	1 (5.0%)	0 (0%)	0.999

rhTPO, recombinant human thrombopoietin.

## Discussion

TPO is an endogenous factor that regulates the proliferation and maturation of megakaryocytes and the generation of platelets, which plays a biological role by binding to c-MPL, a specific receptor on the surface of hematopoietic stem cells and megakaryotic progenitor cells and activates the signaling pathway of Janus kinase 2 (JAK2)/signal transducer and activator of transcription 3 (STAT3) (21, 25–27). In this proof-of-concept study, patients newly diagnosed with LR-MDS were enrolled. The baseline characteristics of the two groups were compatible. Our study showed that the addition of rhTPO can accelerate the recovery of platelets for the first 2 months of treatment and decreased the platelet transfusion rate at the early stage of treatment compared with the control group, which can be verified by studies focused on other thrombocytopenia diseases, i.e., studies on ITP or AA, which we mentioned previously (22, 28).

For newly diagnosed LR-MDS, short-term treatment with rhTPO can benefit platelets for the first 2 months, with the benefit disappearing after 3 months. This is probably due to the relatively short treatment duration of rhTPO, which might be solved by prolonged rhTPO treatment. However, since rhTPO has to be administered subcutaneously, it is not very convenient for long-term use. Some TPO-RAs such as EPAG have shown effectiveness in LR-MDS. Administration can be changed to oral TPO-RAs if patients do not respond well. However, it should also be noted that rhTPO has different binding sites and may have other subtle mechanistic differences from other TPO-RAs (29, 30). It may also not have long-term maintenance effects, as observed in other TPO-RAs.

Nevertheless, this was meaningful in clinical practice because most of the bleeding happened at the early time after diagnosis when other treatments for MDS did not work yet. Collectively, these findings suggested that the addition of rhTPO can accelerate the early platelet response in patients with LR-MDS, but this benefit did not transform to overall response at different time points of the study. The hematopoietic stimulation of TPO-RAs such as EPAG or romiplostim in patients with AA or MDS had been well investigated (31).

In terms of therapy response, 55% and 47% of MDS patients achieved platelet response after romiplostim or EPAG therapy at 4 or 16 weeks, respectively (15, 16). Furthermore, unilineage and bilineage responses were observed in 46% (5/11) and 55% (6/11) of patients with low–intermediate risk-1 MDS at 16 weeks after the start of EPAG treatment (32). This may be explained by the fact that c-MPL exists on the surface of various hematopoietic cells, including hematopoietic stem cells and hematopoietic progenitor cells of various lines (25, 33–35). Unlike other TPO-RAs, rhTPO mainly induces megakaryocyte development and platelet production and has less effect on erythroid and granulocyte differentiation (26, 27), which illustrated our findings for platelet response only. Another concern is the short-term treatment of rhTPO. We devised the 7 days per month for 3 months regimen because most of the patients cannot tolerate long-term injection per month. However, in the present study, we also observed the tendency of higher neutrophil counts at 1–3 months after rhTPO therapy, although not statistically significant. The dose-dependent effects on hematologic response, like those observed with EPAG or romiplostim, may need future experiments with a higher dose or longer-term administration of rhTPO.

Another concern for TPO-RAs was the clone evolution. As reported previously, the trial was stopped because of concerns related to excess blasts and progression to AML in the romiplostim arm, but the 5-year follow-up did not demonstrate an increased risk of AML or death (16, 17). However, a transient elevation of circulating blasts was observed in ~10% of patients, for which close monitoring was recommended, as well as avoidance of TPO-RA use in MDS patients with excess blasts (>5%). To avoid the possible transformation to high-risk MDS or AML, we carefully excluded those with excess blasts, although one patient in the rhTPO group developed deletion 7q chromosome abnormality and had myeloblast increase from <2% to 5% at 9 months. −7/del (7q), a well-known frequent occurrence in MDS patients, had been reported in a LR-MDS patient after treatment with EPAG (31). Although the number was too low to draw any conclusions, close monitoring and a longer follow-up time are needed in the future. As for disease progression, more platelet deterioration was noted in the non-rhTPO group, although not significant. In addition, bone fibrosis was not observed in our study, as verified by other studies showing that rhTPO did not increase transformation to MDS and the degree of fibrosis in patients with AA (21, 25).

As for the side effects, consistent with the adverse event profiles reported by rhTPO-related studies (21, 25), the adverse events in this study were mild and recoverable from with or without medications. ICH was the most common life-threatening bleeding complication in patients (36), which occurred in one patient who did not respond to rhTPO when the platelet count was 2 × 10^9^/L. This accidentally serious adverse event highlighted the importance of the prevention of bleeding-related adverse events as early as possible in patients who are resistant to rhTPO. Thromboembolic risk is another important aspect that needs special attention in treatment with TPO-RAs. An updated systematic review revealed that the use of eltrombopag or romiplostim was associated with a higher risk of thromboembolic events (relative risk = 1.81, 95% CI = 1.04–3.14) in adult thrombocytopenic patients (37). With regard to rhTPO, we did not notice thromboembolic events, so far; however, the dose of rhTPO should be carefully adjusted in rapid responders, especially for elderly patients, and careful monitoring for such events is mandatory.

In terms of the interaction between stanozolol and rhTPO, there are no publications so far on the interactions between these two drugs; however, as shown in our previous study, stanozolol can improve erythropoiesis through the erythropoietin (EPO) and erythropoietin receptor (EPOR) pathways and regulate immune dysfunction in patients with AA (38). It does not change the level of TPO in patients with AA. Therefore, it probably does not affect the action of rhTPO in LRM-MDS patients.

There are some unavoidable limitations in our study. Firstly, some of the patients in the non-rhTPO group withdrew at the early stage and were lost to follow-up. This can cause bias because these patients may have had poor response or had serious complications that we did not know about. Secondly, this prospective cohort enrolled only 35 eligible patients; therefore, some of the findings can be underestimated due to the small sample size. Finally, for the rhTPO group, rhTPO was administered at a fixed dosage, which may have resulted to a higher false-negative rate in the rhTPO group because patients who did not react to the lower dose of rhTPO may respond to a higher dose, as observed in other TPO-RAs. Even with these limitations, our results, for the first time, indicated that rhTPO was effective and well tolerated, with no evidence of increasing clone evolution for patients with LR-MDS. Further prospective clinical trials with a higher rhTPO dose and a longer follow-up in a larger patient cohort might be valuable.

## Data Availability Statement

The raw data supporting the conclusions of this article is available by contacting the correspondence author.

## Ethics Statement

The studies involving human participants were reviewed and approved by the Institutional Ethics Committee of Peking Union Medical College Hospital. The patients/participants provided written informed consent to participate in this study.

## Author Contributions

YY and BH were responsible for the initial plan and study design. YY and JJ were for responsible data collection. YY and TZW performed data analyses and data interpretation. YY drafted the manuscript. ZT, CY, and MC participated in data interpretation. YY and BH are guarantors and had full access to all of the data, including statistical reports and tables, and take full responsibility for the integrity of the data and the accuracy of the data analysis. All authors, external and internal, had full access to all of the data (including statistical reports and tables) in the study and can take responsibility for the integrity of the data and the accuracy of the data analysis, as well as independent of the funders. All authors contributed to the article and approved the submitted version.

## Funding

This study was supported by grants from Beijing Natural Science Foundation (7192168), the Non-profit Central Research Institute Fund of Chinese Academy of Medical Sciences (2019XK 320047), the National Key Research and Development Program of China (2016YFC0901500), and the National Natural Science Foundation of China (81970106). The funders played no role in the study design, the collection, analysis, and interpretation of data, the writing of the report, and the decision to submit the article for publication.

## Conflict of Interest

The authors declare that the research was conducted in the absence of any commercial or financial relationships that could be construed as a potential conflict of interest.

## Publisher’s Note

All claims expressed in this article are solely those of the authors and do not necessarily represent those of their affiliated organizations, or those of the publisher, the editors and the reviewers. Any product that may be evaluated in this article, or claim that may be made by its manufacturer, is not guaranteed or endorsed by the publisher.

## Abbreviations

LR-MDS, lower risk myelodysplastic syndrome; rhTPO, recombinant human thrombopoietin; AML, acute myeloid leukemia; TPO-RAs, thrombopoietin receptor agonists; ITP, immune thrombocytopenia; ANC, absolute neutrophil count; IWG, International Working Group; LDH, lactate dehydrogenase; SF, serum ferritin; ICH, intracranial hemorrhage; SAA, severe aplastic anemia; PNH, paroxysmal nocturnal hemoglobinuria; IPSS-R, Revised International Prognostic Scoring System; ALT, alanine transaminase; AST, aspartate transaminase; TBIL, total bilirubin; EPAG, Eltrombopag.

## References

[1] Arber DA Orazi A Hasserjian R Thiele J Borowitz MJ Le Beau MM. The 2016 Revision to the World Health Organization Classification of Myeloid Neoplasms and Acute Leukemia. Blood (2016) 127(20):2391–405. doi: 10.1182/blood-2016-03-643544 27069254

[2] Patnaik MM Tefferi A. Myelodysplastic Syndromes With Ring Sideroblasts (MDS-RS) and MDS/Myeloproliferative Neoplasm With RS and Thrombocytosis (MDS/MPN-RS-T) - “2021 Update on Diagnosis, Risk-Stratification, and Management”. Am J Hematol (2021) 96(3):379–94. doi: 10.1002/ajh.26090 33428785

[3] Carraway HE Saygin C. Therapy for Lower-Risk MDS. Hematol Am Soc Hematol Educ Program (2020) 2020(1):426–33. doi: 10.1182/hematology.2020000127 PMC7727572 33275714

[4] Greenberg PL Tuechler H Schanz J Sanz G Garcia-Manero G Sole F. Revised International Prognostic Scoring System for Myelodysplastic Syndromes. Blood (2012) 120(12):2454–65. doi: 10.1182/blood-2012-03-420489 PMC4425443 22740453

[5] van Spronsen MF Ossenkoppele GJ Westers TM van de Loosdrecht AA. Prognostic Relevance of Morphological Classification Models for Myelodysplastic Syndromes in an Era of the Revised International Prognostic Scoring System. Eur J Cancer (2016) 56:10–20. doi: 10.1016/j.ejca.2015.12.004 26798967

[6] Pagliuca S Gurnari C Visconte V. Molecular Targeted Therapy in Myelodysplastic Syndromes: New Options for Tailored Treatments. Cancers (Basel) (2021) 13(4):784. doi: 10.3390/cancers13040784 33668555 PMC7917605

[7] Goel R Josephson CD Patel EU Petersen MR Packman Z Gehrie E. Individual- and Hospital-Level Correlates of Red Blood Cell, Platelet, and Plasma Transfusions Among Hospitalized Children and Neonates: A Nationally Representative Study in the United States. Transfusion (2020) 60(8):1700–12. doi: 10.1111/trf.15855 PMC7951993 32589286

[8] Kantarjian H Giles F List A Lyons R Sekeres MA Pierce S. The Incidence and Impact of Thrombocytopenia in Myelodysplastic Syndromes. Cancer (2007) 109(9):1705–14. doi: 10.1002/cncr.22602 17366593

[9] Nachtkamp K Stark R Strupp C Kundgen A Giagounidis A Aul C. Causes of Death in 2877 Patients With Myelodysplastic Syndromes. Ann Hematol (2016) 95(6):937–44. doi: 10.1007/s00277-016-2649-3 27025507

[10] Platzbecker U Steensma DP Van Eygen K Raza A Santini V Germing U. Imerge: A Study to Evaluate Imetelstat (GRN163L) in Transfusion-Dependent Subjects With IPSS Low or Intermediate-1 Risk Myelodysplastic Syndromes (MDS) That Is Relapsed/Refractory to Erythropoiesis-Stimulating Agent (ESA) Treatment. Blood (2019) 134(supp 1):4248. doi: 10.1182/blood-2019-127435

[11] Banu N Wang JF Deng B Groopman JE Avraham H. Modulation of Megakaryocytopoiesis by Thrombopoietin: The C-Mpl Ligand. Blood (1995) 86(4):1331–8. doi: 10.1182/blood.V86.4.1331.bloodjournal8641331 7632939

[12] Panse J. Diagnosis and Therapy of Aplastic Anemia - Update 2021. Dtsch Med Wochenschr (2021) 146(7):451–4. doi: 10.1055/a-1169-0902 33780990

[13] Provan D Arnold DM Bussel JB Chong BH Cooper N Gernsheimer T. Updated International Consensus Report on the Investigation and Management of Primary Immune Thrombocytopenia. Blood Adv (2019) 3(22):3780–817. doi: 10.1182/bloodadvances.2019000812 PMC6880896 31770441

[14] Kuter DJ. The Biology of Thrombopoietin and Thrombopoietin Receptor Agonists. Int J Hematol (2013) 98(1):10–23. doi: 10.1007/s12185-013-1382-0 23821332

[15] Kantarjian H Fenaux P Sekeres MA Becker PS Boruchov A Bowen D. Safety and Efficacy of Romiplostim in Patients With Lower-Risk Myelodysplastic Syndrome and Thrombocytopenia. J Clin Oncol (2010) 28(3):437–44. doi: 10.1200/JCO.2009.24.7999 20008626

[16] Oliva EN Alati C Santini V Poloni A Molteni A Niscola P. Eltrombopag *Versus* Placebo for Low-Risk Myelodysplastic Syndromes With Thrombocytopenia (Eqol-MDS): Phase 1 Results of a Single-Blind, Randomised, Controlled, Phase 2 Superiority Trial. Lancet Haematol (2017) 4(3):e127–e36. doi: 10.1016/S2352-3026(17)30012-1 28162984

[17] Giagounidis A Mufti GJ Fenaux P Sekeres MA Szer J Platzbecker U. Results of a Randomized, Double-Blind Study of Romiplostim Versus Placebo in Patients With Low/Intermediate-1-Risk Myelodysplastic Syndrome and Thrombocytopenia. Cancer (2014) 120(12):1838–46. doi: 10.1002/cncr.28663 PMC4298760 24706489

[18] Kuter DJ Goodnough LT Romo J DiPersio J Peterson R Tomita D. Thrombopoietin Therapy Increases Platelet Yields in Healthy Platelet Donors. Blood (2001) 98(5):1339–45. doi: 10.1182/blood.V98.5.1339 11520780

[19] Liu XG Bai XC Chen FP Cheng YF Dai KS Fang MY. Chinese Guidelines for Treatment of Adult Primary Immune Thrombocytopenia. Int J Hematol (2018) 107(6):615–23. doi: 10.1007/s12185-018-2445-z 29619624

[20] Wang S Yang R Zou P Hou M Wu D Shen Z. A Multicenter Randomized Controlled Trial of Recombinant Human Thrombopoietin Treatment in Patients With Primary Immune Thrombocytopenia. Int J Hematol (2012) 96(2):222–8. doi: 10.1007/s12185-012-1124-8 22753022

[21] Yu Y Wang M Hou Y Qin P Zeng Q Yu W. High-Dose Dexamethasone Plus Recombinant Human Thrombopoietin *vs.* High-Dose Dexamethasone Alone as Frontline Treatment for Newly Diagnosed Adult Primary Immune Thrombocytopenia: A Prospective, Multicenter, Randomized Trial. Am J Hematol (2020) 95(12):1542–52. doi: 10.1002/ajh.25989 32871029

[22] Zhou H Xu M Qin P Zhang HY Yuan CL Zhao HG. A Multicenter Randomized Open-Label Study of Rituximab Plus Rhtpo *vs.* Rituximab in Corticosteroid-Resistant or Relapsed ITP. Blood (2015) 125(10):1541–7. doi: 10.1182/blood-2014-06-581868 PMC4351949 25575541

[23] Cheson BD Greenberg PL Bennett JM Lowenberg B Wijermans PW Nimer SD. Clinical Application and Proposal for Modification of the International Working Group (IWG) Response Criteria in Myelodysplasia. Blood (2006) 108(2):419–25. doi: 10.1182/blood-2005-10-4149 16609072

[24] Common Terminology Criteria for Adverse Events (CTCAE) Version 5. (2017) US Department of Health and Human Services, National Institutes of Health, National Cancer Institute.

[25] Wang H Dong Q Fu R Qu W Ruan E Wang G. Recombinant Human Thrombopoietin Treatment Promotes Hematopoiesis Recovery in Patients With Severe Aplastic Anemia Receiving Immunosuppressive Therapy. BioMed Res Int (2015) 2015:597293. doi: 10.1155/2015/597293 25861635 PMC4377357

[26] Le K Wellik LE Maurer MJ McPhail ED Witzig TE Gupta M. JAK2 Activation Promotes Tumorigenesis in ALK-Negative Anaplastic Large Cell Lymphoma *via* Regulating Oncogenic STAT1-PVT1 Lncrna Axis. Blood Cancer J (2021) 11(3):56. doi: 10.1038/s41408-021-00447-x 33712566 PMC7955124

[27] Jing FM Zhang XL Meng FL Liu XM Shi Y Qin P. Anti-C-Mpl Antibodies in Immune Thrombocytopenia Suppress Thrombopoiesis and Decrease Response to Rhtpo. Thromb Res (2018) 170:200–6. doi: 10.1016/j.thromres.2018.08.021 30199786

[28] Qian J Cao X Shen Q Cai YF Lu W Yin H. Thrombopoietin Promotes Cell Proliferation and Attenuates Apoptosis of Aplastic Anemia Serum-Treated 32D Cells *via* Activating STAT3/STAT5 Signaling Pathway and Modulating Apoptosis-Related Mediators. Cell Transplant (2021) 30:963689720980367. doi: 10.1177/0963689720980367 33586472 PMC7890722

[29] Linker C Anderlini P Herzig R Christiansen N Somlo G Bensinger W. Recombinant Human Thrombopoietin Augments Mobilization of Peripheral Blood Progenitor Cells for Autologous Transplantation. Biol Blood Marrow Transplant (2003) 9(6):405–13. doi: 10.1016/S1083-8791(03)00101-0 12813449

[30] Erickson-Miller CL DeLorme E Tian SS Hopson CB Stark K Giampa L. Discovery and Characterization of a Selective, Nonpeptidyl Thrombopoietin Receptor Agonist. Exp Hematol (2005) 33(1):85–93. doi: 10.1016/j.exphem.2004.09.006 15661401

[31] Vicente A Patel BA Gutierrez-Rodrigues F Groarke E Giudice V Lotter J. Eltrombopag Monotherapy Can Improve Hematopoiesis in Patients With Low to Intermediate Risk-1 Myelodysplastic Syndrome. Haematologica (2020) 105(12):2785–94. doi: 10.3324/haematol.2020.249995 PMC7716353 33256377

[32] Mittelman M Platzbecker U Afanasyev B Grosicki S Wong RSM Anagnostopoulos A. Eltrombopag for Advanced Myelodysplastic Syndromes or Acute Myeloid Leukaemia and Severe Thrombocytopenia (ASPIRE): A Randomised, Placebo-Controlled, Phase 2 Trial. Lancet Haematol (2018) 5(1):34–43. doi: 10.1016/S2352-3026(17)30228-4 29241762

[33] Matsuda A Misumi M Ishikawa M Yagasaki F Jinnai I Bessho M. Long-Term Improvement of Anaemia in a Patient With Aplastic Anaemia by Short-Term Administration of Pegylated Recombinant Human Megakaryocyte Growth and Development Factor. Br J Haematol (2004) 125(6):818–9. doi: 10.1111/j.1365-2141.2004.04980.x 15180875

[34] Yonemura Y Miyake H Asou N Mitsuya H. Long-Term Efficacy of Pegylated Recombinant Human Megakaryocyte Growth and Development Factor in Therapy of Aplastic Anemia. Int J Hematol (2005) 82(4):307–9. doi: 10.1532/IJH97.05032 16298819

[35] Nishiyama U Kuwaki T Akahori H Kato T Ikeda Y Miyazaki H. Decreased Prothrombotic Effects of Pegylated Recombinant Human Megakaryocyte Growth and Development Factor in Thrombocytopenic State in a Rat Thrombosis Model. J Thromb Haemost (2005) 3(2):355–60. doi: 10.1111/j.1538-7836.2005.01113.x 15670044

[36] Melboucy-Belkhir S Khellaf M Augier A Boubaya M Levy V Le Guenno G. Risk Factors Associated With Intracranial Hemorrhage in Adults With Immune Thrombocytopenia: A Study of 27 Cases. Am J Hematol (2016) 91(12):499–501. doi: 10.1002/ajh.24529 27528011

[37] Catala-Lopez F Corrales I de la Fuente-Honrubia C Gonzalez-Bermejo D Martin-Serrano G Montero D. Risk of Thromboembolism With Thrombopoietin Receptor Agonists in Adult Patients With Thrombocytopenia: Systematic Review and Meta-Analysis of Randomized Controlled Trials. Med Clin (Barc) (2015) 145(12):511–9. doi: 10.1016/j.medcli.2015.03.014 26051432

[38] Li H Long Z Wang T Han B. Stanozolol and Danazol Have Different Effects on Hematopoiesis in the Murine Model of Immune-Mediated Bone Marrow Failure. Front Med (Lausanne) (2021) 8:615195. doi: 10.3389/fmed.2021.615195 34124083 PMC8193361

